# Evaluation of efficacy and toxicity of nivolumab combined with or without docetaxel in patients with advanced NSCLC

**DOI:** 10.1007/s00262-021-02964-x

**Published:** 2021-06-15

**Authors:** Yang Wang, Jun Nie, Ling Dai, Weiheng Hu, Jie Zhang, Xiaoling Chen, Xiangjuan Ma, Guangming Tian, Jindi Han, Sen Han, Di Wu, Jieran Long, Ziran Zhang, Jian Fang

**Affiliations:** Key Laboratory of Carcinogenesis and Translational Research (Ministry of Education/Beijing), Department of Thoracic Oncology II, Peking University Cancer Hospital & Institute, Haidian District, 52# Fucheng Road, Beijing, 100142 China

**Keywords:** Non-small cell Lung cancer, Nivolumab, Docetaxel, Combination therapy, EGFR, ALK

## Abstract

**Background:**

The combination of PD-1/PD-L1 inhibitor and chemotherapy has been clinically confirmed to be beneficial as the first-line treatment of patients with advanced NSCLC. This study aimed to assess the effect of nivolumab + docetaxel versus nivolumab monotherapy in patients with NSCLC after the failure of platinum doublet chemotherapy.

**Materials and methods:**

The efficacy and toxicity of nivolumab + docetaxel combination therapy versus nivolumab monotherapy were compared in this retrospective study. Primary endpoint of the study was progression-free survival (PFS), and the secondary endpoints were objective response rate (ORR), overall survival (OS), and toxicity.

**Results:**

Between November 2017 and December 2019, 77 patients were included in this study, with 58 patients in the nivolumab group and 19 in the nivolumab + docetaxel group. The median follow-up was 18 months, and the PFS was 8 months for patients receiving nivolumab + docetaxel and 2 months for those receiving nivolumab alone (*p* = 0.001), respectively. Nivolumab + docetaxel showed superior OS compared with nivolumab, with the median OS unreached versus 7 months (*p* = 0.011). Among patients without EGFR/ALK variation, compared to nivolumab monotherapy, nivolumab + docetaxel showed better PFS (*p* = 0.04) and OS (*p*  = 0.05). There was no significant difference in grade 3–4 adverse events (AEs) between the two groups (*p* = 0.253).

**Conclusions:**

The combination of nivolumab and docetaxel demonstrated a meaningful improvement in progression-free survival and overall survival compared to nivolumab monotherapy, in patients with NSCLC after the failure of platinum doublet chemotherapy, irrespective of EGFR/ALK variation status.

## Introduction

Lung cancer is the most common malignant tumor and the leading cause of cancer-related deaths worldwide [1]. Among the lung cancer subtypes, non-small cell lung cancer (NSCLC) is most prevalent. Immunotherapy has profoundly changed the treatment modalities and outcomes of advanced NSCLC, as well as significantly improved the tumor response and overall survival (OS). Hence, researchers continue to explore different combinations of immunotherapy, including dual immunotherapy combination, immunotherapy combined with chemotherapy, immunotherapy combined with targeted therapy, and immunotherapy combined with anti-vascular drugs, in patients with advanced NSCLC. The KEYNOTE-189 and KEYNOTE-407 studies confirmed the survival advantage of pembrolizumab combined with doublet platinum-based chemotherapy as the first-line treatment for NSCLC, compared with doublet platinum-based chemotherapy [2, 3]. Similarly, IMPOWER150 and IMPOWER130 studies demonstrated superior survival with atezolizumab combined with chemotherapy in advanced NSCLC [4, 5].

Combination of PD-1/PD-L1 inhibitor and platinum doublet chemotherapy brought significant improvements in survival of patients with advanced NSCLC. Several studies have shown that chemotherapy could increase the infiltration of CD8 + T cells and down-regulate the regulatory T cells (Tregs) and convert the non-immunogenic microenvironment to immunogenic, thereby enhancing the activity of immunotherapy [6, 7]. Preclinical and clinical studies have established the effectiveness and safety of combined PD-1/PD-L1 inhibitor and chemotherapy as the first-line therapy for advanced NSCLC, which is the preferred treatment option in clinical practice.

As the second-line systemic therapy for NSCLC, nivolumab showed significant improvement in OS versus docetaxel in patients with advanced NSCLC, in both non-squamous and squamous histology, regardless of PD-L1 expression [8, 9]. However, nivolumab often had a slow onset of efficacy, with the median time to response of 2.2 months in squamous NSCLC and 2.1 months in non-squamous NSCLC. In clinical practice, nivolumab was not effective in many patients before deterioration of their general health status or disease progression, thus leading to discontinuation of nivolumab treatment. In addition, nivolumab did not show a significant advantage in progression-free survival (PFS) compared to docetaxel, with a median PFS of 3.5 versus 2.8 months in squamous NSCLC and 2.3 versus 4.3 months in non-squamous NSCLC [10]. Hence, it is important to assess if a similar synergistic activity as in the first-line therapy can be achieved by adding docetaxel to the standard treatment of nivolumab for the second-line therapy of advanced NSCLC. The objective of this retrospective study was to assess the effect of nivolumab + docetaxel combination therapy versus nivolumab monotherapy in patients with NSCLC after the failure of platinum doublet chemotherapy.

## Materials and methods

Patients with stage IIIB to IV NSCLC, who had received nivolumab or nivolumab + docetaxel treatment after the failure of platinum-based chemotherapy at the Department of Thoracic Oncology, Peking University Cancer Hospital and Institute between November 2017 and December 2019, were included in this study. Patients with EGFR/ALK mutation-positive tumors were included if they had progression during or after at least one approved EGFR/ALK inhibitor and platinum-based doublet chemotherapy. Patients who had a poor Eastern Cooperative Oncology Group (ECOG) performance status (ECOG ≥ 3) had participated in clinical trials or received more than four lines of systemic therapy were excluded. Patients’ data, including clinicopathological characteristics, treatment, and follow-up, were extracted from a prospectively maintained database for NSCLC at the Peking University Cancer Hospital and Institute. All the patients provided informed consent with a signature confirmation before treatment. This study was approved by the Ethics Committee of the Peking University Cancer Hospital and Institute.

Nivolumab (3 mg/kg) was given every two weeks in the nivolumab monotherapy group. In the nivolumab + docetaxel group, at the initial phase of nivolumab treatment, all the patients received 4–6 cycles of docetaxel according to the investigator’s choice, and docetaxel (75 mg/m^2^) was given every three weeks. During the cycles combined with docetaxel, nivolumab (3 mg/kg) was delivered every three weeks in order to maintain consistency with the chemotherapy and reduce the number of hospitalizations. After completion of induction chemotherapy, nivolumab (3 mg/kg) was given every 2 weeks until disease progression or unacceptable toxicity. The PD-L1 expression was tested using PD‐L1 IHC 22C3 pharmDx assay.

The primary endpoint of the study was PFS, and the secondary endpoints were objective response rate (ORR), OS, and the incidence and severity of adverse events (AEs). Best overall response (BOR) and disease control rate (DCR) were also evaluated. The PFS was defined as the time from the start of nivolumab or nivolumab + docetaxel treatment until disease progression or death, while OS was defined from the start of nivolumab or nivolumab + docetaxel until death. The tumor responses of patients were assessed by computed tomography (CT) or magnetic resonance imaging (MRI) every 6–8 weeks after starting treatment, according to the Response Evaluation Criteria in Solid Tumor (V1.1). ORR was defined as the proportion of patients achieving complete response (CR) or partial response (PR). DCR was defined as the number of patients achieving CR, PR or stable disease.

Chi-square tests or Fisher’s exact tests were used to analyze the statistical significance of categorical variables in the two groups. Independent sample t test was used for analyzing the difference between continuous variables. The difference of ORR and DCR were calculated using two separate chi-square tests (Pearson’s chi-square test and Yates’s correction for continuity, respectively). PFS and OS were analyzed by Kaplan–Meier survival curves. Differences were determined to be statistically significant if *p* value < 0.05. Graphs were plotted by GraphPad Prism software, and data were analyzed by SPSS and GraphPad Prism.

## Results

### Patient characteristics

A total of 77 patients were enrolled in this retrospective study between November 2017 and December 2019, with 58 patients in the nivolumab group and 19 in the nivolumab + docetaxel group. The baseline characteristics (Table 1), including gender, age, histology, stage at diagnosis, ECOG status, EGFR/ALK variation status, PD-L1 expression, and brain metastasis, were generally similar between the two groups, although the therapeutic lines when receiving nivolumab monotherapy or combination therapy had a significant difference between the two groups (*p* = 0.02). A total of nine patients (9/77, 11.7%) with EGFR (7/77, 9.1%) or ALK variations (2/77, 2.6%) were included in this study (seven patients with EGFR variation and two with ALK variation). All nine patients with sensitizing EGFR or ALK mutations received corresponding targeted therapy before platinum-based chemotherapy. Only 37 of 77 patients (46.6%) underwent the PD-L1 expression test, and the proportion of PD-L1-positive patients was similar between the two groups.

**Table 1:** Patient baseline characteristics in nivolumab group and nivolumab combined docetaxel group

Variable	All patients *n* = 77 (%)	Nivolumab *n* = 58 (%)	Nivolumab + Docetaxel *n* = 19 (%)	*p* value
Age (years, median)	62	63	60	0.697
≥ 65	29 (37.7)	23 (39.7)	6 (31.6)	0.582
Gender, man	54 (70.1)	40 (69)	24 (73.7)	
Histology				
Non-squamous	39 (50.6)	28 (48.3)	11 (57.9)	0.467
Squamous	38 (49.4)	30 (51.7)	8 (42.1)	
ECOG				
0–1	67 (87)	49 (84.5)	18 (94.7)	0.436
2	10 (13)	9 (15.5)	1 (5.3)	
Stage at diagnosis				
IIIb–IIIc	3 (3.9)	3 (5.2)	0	0.571
IV	74 (96.1)	55 (94.8)	19 (100%)	
PD-L1 expression				
Positive	22 (28.6)	16 (27.6)	6 (31.6)	0.899
Negative	15 (19.5)	11 (19)	4 (21.1)	
Undetected	40 (51.9)	31 (53.3)	9 (47.4)	
Oncotarget variation				
EGFR/ALK positive	9 (11.7)	7 (12.1)	2 (10.5)	0.856
Therapeutic lines				
Second line	42 (54.5)	25 (43.1)	17 (89.5)	0.002
Third line	27 (35.1)	25 (43.1)	2 (10.5)	
Fourth line	8 (10.4)	8 (13.8)	0	
Brain metastasis				
Yes	54 (70.1)	41 (70.7)	13 (68.4)	0.851
No	23 (29.9)	17 (29.3)	6 (31.6)	

The median number of cycles of nivolumab administered was 5 (range 1–37) in the nivolumab group and 6 (range 2–18) in the nivolumab + docetaxel group. The median follow-up in the nivolumab group was 19 months (range 1–42), and 8 of 58 patients (13.7%) were still receiving the nivolumab treatment. The median follow-up in the nivolumab + docetaxel group was 11 months (range 1–22), and 8 of 19 patients (42.1%) were still receiving the nivolumab treatment. Meanwhile, 26 of 58 patients (44.8%) in the nivolumab group and 10 of 18 patients (55.6%) in the nivolumab + docetaxel group had received subsequent therapy, and the most common therapy was anlotinib (a small molecule target-multiple tyrosine kinase inhibitor, which was approved for use in third-line therapy of NSCLC in China). Nineteen patients (52.8%) received anlotinib as the subsequent therapy. Other subsequent therapy included pemetrexed (5 patients, 13.9%), docetaxel (4 patients, 11.1%), S-1 (4 patients, 11.1%), and nab-paclitaxel (4 patients, 11.1%).

### Efficacy

**Fig. 1:**
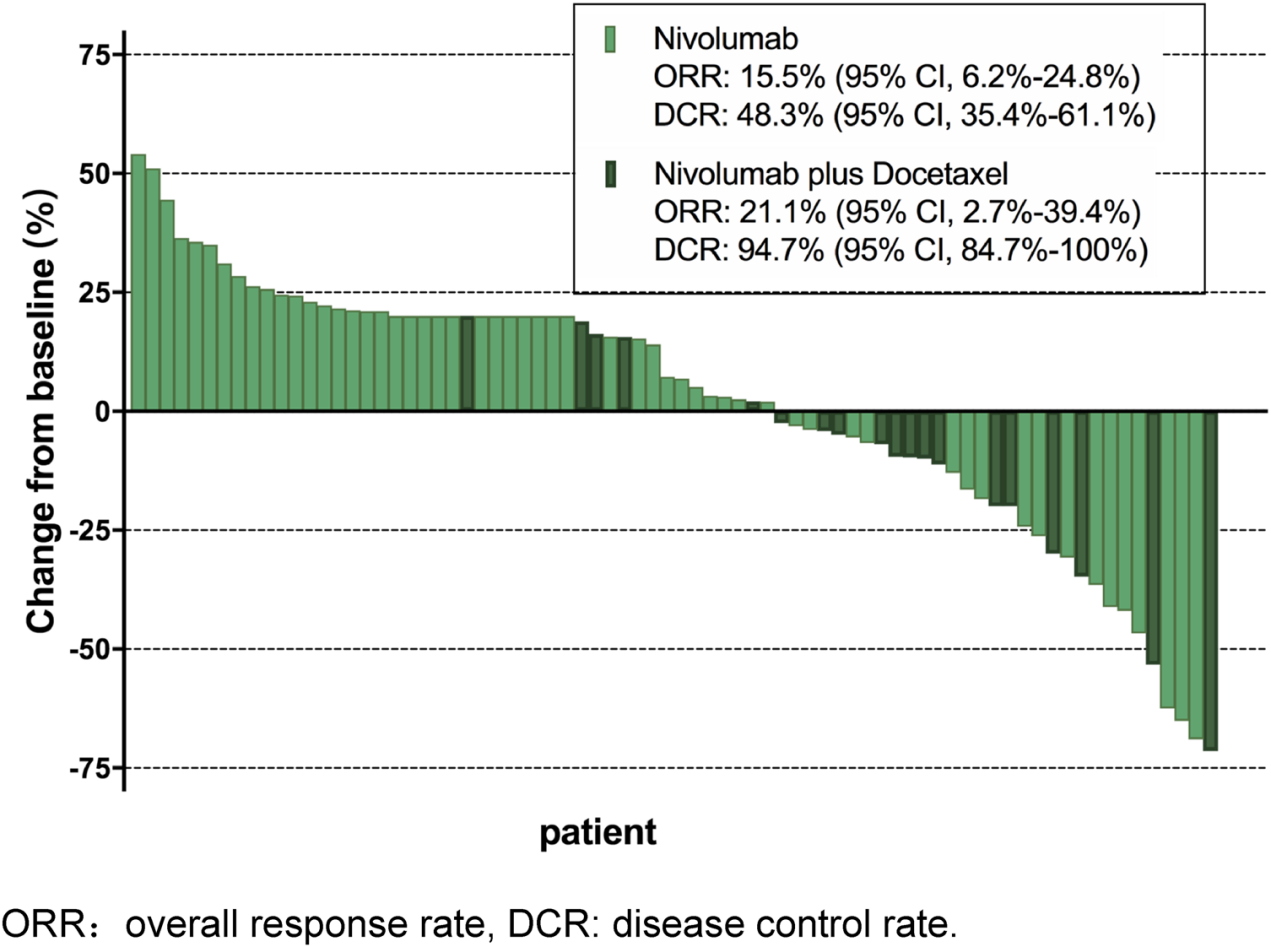
Best change in target lesions from baseline for all population. ORR, overall response rate; DCR, disease control rate

Best change in target lesions from baseline for all participants is listed in Fig. 1. The ORR was 15.5% (95% CI 6.2–24.8%) vs. 21.1% (95% CI 2.7–39.4%) (*p* = 0.576) in the nivolumab group vs. the nivolumab + docetaxel group, respectively. No patient achieved CR. The DCR was 48.3% (95% CI 35.4–61.1%) in the nivolumab group and 94.7% (95% CI 84.7–100%) in the nivolumab + docetaxel group (*p* < 0.001). Among patients with EGFR-wild-type and ALK negative, DCR was 48.3% (95% CI 35.4–61.1%) in the nivolumab group and 94.7% (95% CI 84.7–100%) in the nivolumab + docetaxel group (*p* < 0.001), but ORR did not show statistical difference between the two groups (*p* = 0.477).

**Fig. 2:**
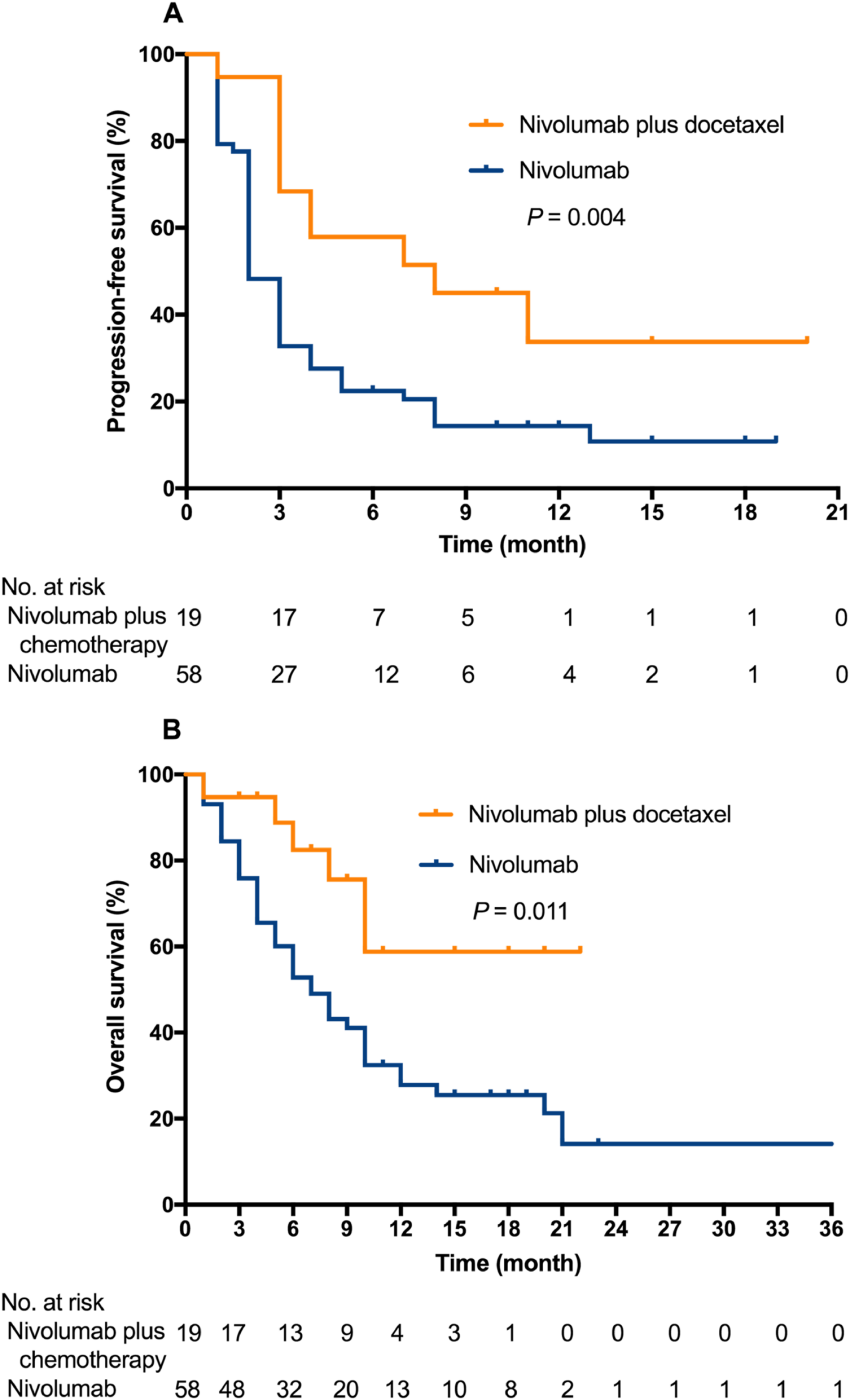
Kaplan–Meier curves for progression-free survival (**A**) and overall survival (**B**) for all patients

The nivolumab + docetaxel group had a significantly prolonged PFS compared with nivolumab alone (8 months; 95% CI 1.2–14.7; vs. 2 months; 95% CI 1.6–2.4; *p* = 0.001) (Fig. 2A). Nivolumab + docetaxel also showed superior OS compared with nivolumab alone (Fig. 2B), with the median OS unreached in the nivolumab + docetaxel group versus 7 months (95% CI 4.4–9.6) (*p* = 0.011) in the nivolumab group. Improved PFS and OS with nivolumab + docetaxel were also observed in the EGFR/ALK-negative subgroup. Among patients without EGFR/ALK variation, median PFS was 7 months (95% CI 2.4–11.6) with nivolumab + docetaxel and 3 months (95% CI 2.6–3.4) with nivolumab alone (*p* = 0.04); median OS was not reached with nivolumab + docetaxel and 8 months (95% CI 5.0–11.0) with nivolumab alone (*p* = 0.05). For patients with brain metastasis, the nivolumab + docetaxel group had a significantly prolonged PFS compared with nivolumab alone (unreached; vs. 3 months; *p* = 0.03), but the OS did not show survival benefits (*p* = 0.187).

Subgroup analysis showed nivolumab + docetaxel brought benefits in most pre-specified subgroups.

**Fig. 3:**
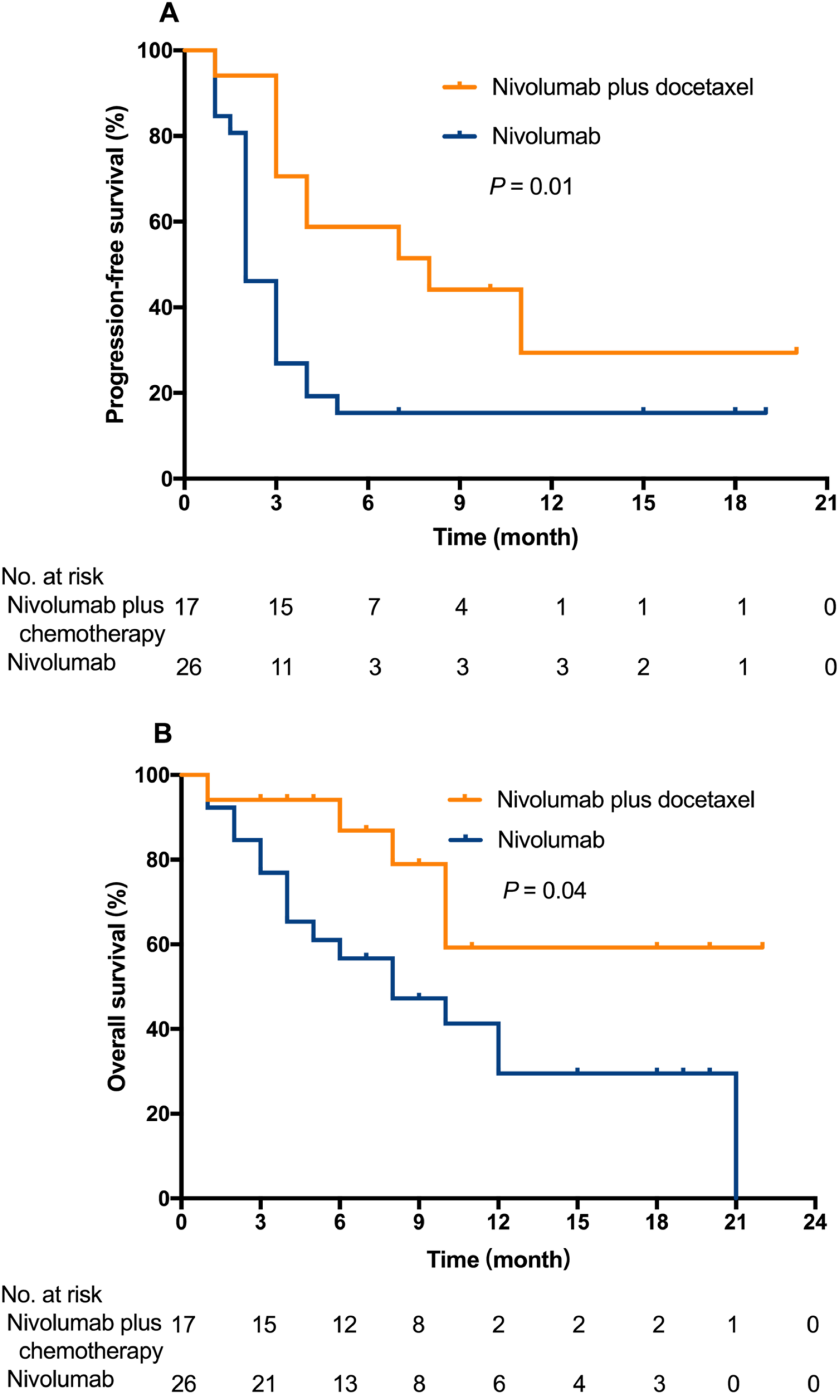
Kaplan–Meier curves for progression-free survival (**A**) and overall survival (**B**) for patients on second-line therapy

Since the therapeutic lines in the two groups were imbalanced, we analyzed the PFS and OS in the second-line therapy subgroup. For patients receiving the second-line therapy, median PFS was 8 months (95% CI 1.2–14.8) with nivolumab + docetaxel and 2 months (95% CI 1.5–2.6) with nivolumab alone (*p* = 0.01, Fig. 3A); median OS was not reached with nivolumab + docetaxel and 8 months (95% CI 2.7–13.2) with nivolumab alone (*p* = 0.04, Fig. 3B). Nivolumab + docetaxel showed a significant advantage for PFS and OS compared with nivolumab monotherapy in second-line therapy. We also compared the PFS and OS in patients with squamous histology vs non-squamous histology. The PFS (*p* = 0.112) and OS (*p* = 0.29) had no significant difference between patients with squamous and non-squamous histology.

### Toxicity

**Fig. 4:**
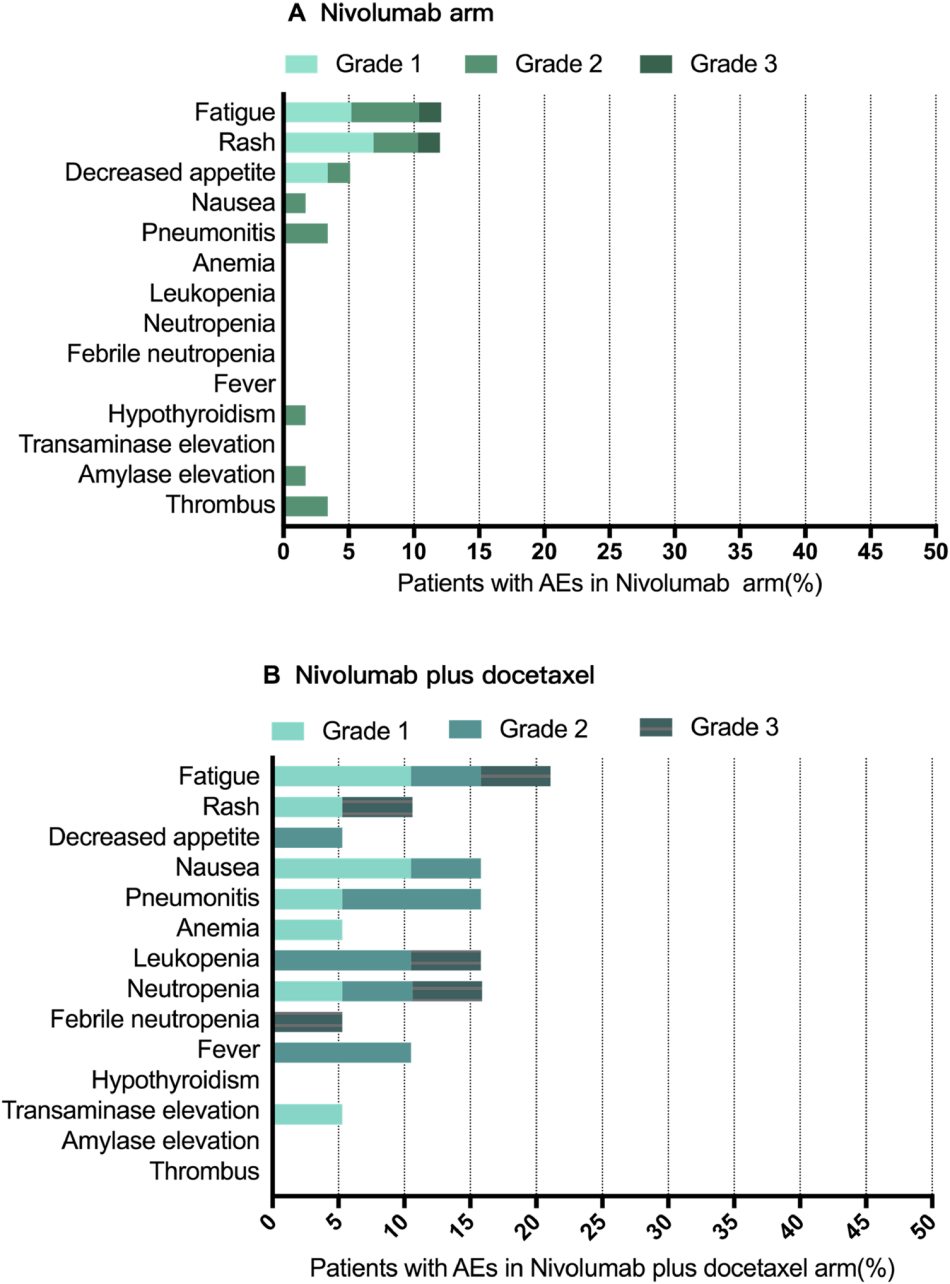
Treatment-related adverse events in patients treated with nivolumab (**A**) or nivolumab plus docetaxel (**B**)

Any-grade adverse events (AEs) were more frequent with nivolumab + docetaxel (14/19, 73.7%) than nivolumab alone (24/58, 41.4%) (*p* = 0.015), but grade 3–4 AEs did not display a significant difference between the two groups (10.5% vs. 3.4%, *p* = 0.253). All-causality, any-grade AEs in the whole cohort are shown in Fig. 4. The most frequent treatment-related AEs in the nivolumab + docetaxel arm included fatigue (21.1%), nausea (15.8%), leukopenia and neutropenia (15.8%), pneumonitis (15.8%), rash (10.5%), fever (10.5%), and decreased appetite (5.3%). In the nivolumab monotherapy group, the most frequent treatment-related AEs included fatigue (12.1%), rash (12.1%), decreased appetite (5.2%), pneumonitis (3.4%), and nausea (1.7%). Of these, the frequencies of nausea, leukopenia and neutropenia were higher in the nivolumab + docetaxel group than in the nivolumab group. Compared to nivolumab alone, nivolumab + docetaxel might increase the incidence of pneumonitis (15.8% vs. 3.4%), though not statistically significant (*p* = 0.174). There was no grade 3–4 pneumonitis in the entire cohort.

The uncommon AEs in the nivolumab + docetaxel group included one patient with grade 1 transaminase elevation. In the nivolumab monotherapy group, one patient developed hypothyroidism, one had asymptomatic amylase elevation, while two patients developed venous thrombosis, which could also be caused by lung cancer, and thus the relation to nivolumab treatment was uncertain. One patient in each group discontinued treatment due to treatment-related AEs, i.e., grade 2 pneumonia. There were no grade 4 toxicities and treatment‐related deaths in the two groups.

## Discussion

According to the results of the CheckMate 017 and CheckMate 057 studies [8, 9], nivolumab treatment has become the new standard second-line or above treatment for NSCLC patients. In two phase III clinical studies, nivolumab showed a longer OS and fewer AEs than docetaxel in squamous and non-squamous NSCLC. The analysis of long-term results confirmed that nivolumab had significant clinical efficacy compared with docetaxel [10, 11]. The ORR of nivolumab was 19% for adenocarcinoma and 20% for squamous cell carcinoma, while ORR of docetaxel was 12%. Nivolumab showed lasting disease remission, but only in limited patients. The median treatment duration in the pooled CheckMate 017/057 studies for nivolumab and docetaxel was 2.8 (range 0–51.8) months and 2.1 (range 0–20.0) months, respectively, which suggested that a considerable number of patients could not benefit from the second-line nivolumab therapy. Therefore, as in the first-line therapy of NSCLC, perhaps more patients could benefit from the combination of nivolumab and chemotherapy. Recently, a phase II study compared the efficacy and safety of pembrolizumab + docetaxel with docetaxel monotherapy in patients with advanced NSCLC who were previously treated with platinum-based chemotherapy [12]. The results showed that the combination of pembrolizumab + docetaxel was safe, and substantially improved ORR and PFS compared with docetaxel. However, no clinical study has directly compared immunotherapy with immunotherapy + chemotherapy in the second-line treatment of NSCLC. Only a small retrospective study indicated that PD-1/PD-L1 inhibitor + nab-paclitaxel showed significantly longer OS and higher response than PD-1/PD-L1 inhibitor monotherapy in second-line and above therapy for NSCLC [13]. However, the majority of patients in the study received third-line and above therapy (65%), and different types of PD-1/PD-L1 inhibitors were included, which were unreported.

To the best of our knowledge, this is the first study to report that the combination of nivolumab and docetaxel was effective and safe in patients with NSCLC progressing after platinum-based chemotherapy, and had an advantage on PFS and OS compared to the standard nivolumab monotherapy. The median PFS of nivolumab monotherapy in this study was 2 months, which was similar to the reported data in clinical studies and daily practice [8, 9, 14–16], and the median PFS of nivolumab combined with docetaxel was significantly longer at 8 months. Similarly, compared with nivolumab alone, the OS of nivolumab + docetaxel was significantly longer (unreached vs. 7 months, *p* = 0.011). The ORR of nivolumab in this study (15.5%) was slightly lower than previous reports (17.6–22.3%) [17–19], perhaps because in this study almost 57% of the patients were receiving nivolumab as a third or fourth line of therapy. Furthermore, it caused the median OS of nivolumab in this study was lower than it was reported in Checkmate 017 and Checkmate 057 [8, 9]. Another reason could be that, in our study, patients with performance status ECOG score comprised 13%, and patients with brain metastasis comprised 29.9%. Although the response rate of the two groups did not show statistical differences (21.1% vs. 15.5%, *p* = 0.576), the DCR of the combination group of nivolumab and docetaxel was significantly better than that of the nivolumab group (94.7% vs. 48.3%, *p* < 0.001). This showed that the combination therapy could enable more patients to receive immunotherapy of nivolumab for a longer time.

The therapeutic lines were imbalanced in the two treatment groups, and the nivolumab group had more patients receiving third-line or fourth-line therapy, suggesting that any adverse biases between the two groups should be ruled out. Thus, we analyzed the PFS and OS in second-line therapy between the two groups. Results showed that in the second-line therapy subgroup, addition of docetaxel to nivolumab demonstrated higher PFS (8 months, 95% CI 1.2–14.8 vs. 2 months, 95% CI 1.5–2.6; *p* = 0.01) and OS (not reached vs. 8 months, 95% CI 2.7–13.2; *p* = 0.04), which indicated that nivolumab + docetaxel was generally applicable in second-line and follow-up treatment.

In this study, in the whole population as well as in the patients without EGFR/ALK variation, nivolumab + docetaxel demonstrated higher PFS and OS, compared with nivolumab monotherapy. Previous studies showed that immune checkpoint inhibitor (ICI) monotherapy had a poor treatment efficacy in advanced NSCLC patients with sensitizing EGFR mutations. In the subgroup analysis, nivolumab, pembrolizumab and atezolizumab showed comparable PFS and OS as docetaxel [9, 20, 21]. However, the combination of ICIs and chemotherapy may bring survival advantage to patients with EGFR mutations. The Impower150 study first reported that the combination of ICI and chemotherapy displayed higher OS versus chemotherapy alone [22]. Similarly, a phase II trial compared pembrolizumab + docetaxel with docetaxel monotherapy in patients with failed platinum-based chemotherapy, and found that the PFS benefit was also maintained in patients with EGFR variations [12]. Currently, several clinical trials (NCT02864251, NCT03515837) are exploring the beneficial effects of immunotherapy in EGFR-mutant populations.

The safety profile of nivolumab + docetaxel was in accordance with the reported AEs related to nivolumab or docetaxel, and no new AEs were observed. There was no significant difference between the two groups in the proportion of grade 3–4 AEs (10.5% in the nivolumab + docetaxel group vs. 3.4% in the nivolumab group, *p* = 0.253). No fatal AEs occurred in the two groups. Notably, the incidence of pneumonitis in the nivolumab + docetaxel group was higher than in the nivolumab monotherapy group (15.8% vs. 3.4%), although not statistically significant. The possible cause of the difference is that pneumonia is a common AE of docetaxel chemotherapy. In a large-scale, real-world analysis of patients with NSCLC who received docetaxel as second-line therapy, atypical pneumonia was the third most common AE, recorded in 18% of patients, and 10–12% of patients needed emergency visits or treatment [23]. The frequency of pneumonitis previously observed with nivolumab monotherapy ranged from 1 to 10.8% in clinical trials and real-world studies [9, 10, 14, 15, 24, 25]. The age, ECOG status, and pre-existing pulmonary conditions of patients included in the studies affected the frequency of treatment-related pneumonitis. Given the low number of cases analyzed in the nivolumab + docetaxel group, the incidence of pneumonia with combined treatment needs further confirmation.

This study had several limitations. First, the non-randomized and retrospective study design led to unavoidable survival and selection biases. We are currently recruiting patients for a prospective, randomized controlled trial to compare the efficacy and safety of nivolumab monotherapy with nivolumab + docetaxel in advanced NSCLC patients with failed platinum-based chemotherapy. Second, the status of PD-L1 expression was undetected in most patients so we could not perform subgroup analysis with different PD-L1 expression levels. It is not necessary to test PD-L1 expression before nivolumab treatment. Third, the number of patients in this study was small, especially in the nivolumab combined chemotherapy group.

In summary, this retrospective study showed that the combination of nivolumab and docetaxel demonstrated a meaningful improvement in progression-free survival and overall survival, in patients with NSCLC after the failure of platinum doublet chemotherapy, irrespective of EGFR/ALK variation status. This suggested that the combination of immunotherapy and chemotherapy could be a potential treatment choice in NSCLC second-line therapy. As the nivolumab often had a slow onset of efficacy in real-world practice, and the hyperprogressions made patients had little opportunity to receive the third or fourth line of therapies because of bad general conditions. The hyperprogression has an incidence of 6–43% in ICIs monotherapy and has not been reported in combination therapy of ICIs and chemotherapy [26]. The attempts to combine the nivolumab and chemotherapy in second-line therapy in NSCLC patients are clinically significant. Prospective randomized controlled study is needed.

## Data Availability

All data generated or analyzed during this study are included in this published article.
